# Safety and Feasibility of Transcranial Direct Current Stimulation for Cognitive Rehabilitation in Patients With Mild or Major Neurocognitive Disorders: A Randomized Sham-Controlled Pilot Study

**DOI:** 10.3389/fnhum.2019.00273

**Published:** 2019-09-06

**Authors:** Takuma Inagawa, Yuma Yokoi, Zui Narita, Kazushi Maruo, Mitsutoshi Okazaki, Kazuyuki Nakagome

**Affiliations:** ^1^Department of Psychiatry, National Center of Neurology and Psychiatry, National Center Hospital, Kodaira, Japan; ^2^Department of Psychiatry and Behavioral Sciences, Johns Hopkins University School of Medicine, Baltimore, MD, United States; ^3^Faculty of Medicine, University of Tsukuba, Tsukuba, Japan

**Keywords:** transcranial direct current stimulation, cognitive training, neurocognitive disorder, dementia, mild cognitive impairment

## Abstract

**Introduction:**

Transcranial direct current stimulation (tDCS) is a potentially novel strategy for cognitive enhancement in patients with mild or major neurocognitive disorders. This study aims to assess the safety and efficacy of tDCS during cognitive training on cognitive functioning in patients with mild or major neurocognitive disorders.

**Methods:**

This study was primarily a single arm for safety, secondary a two-arm, parallel, randomized, and sham-controlled trial for potential efficacy. Patients with mild or major neurocognitive disorders were recruited. The participants and raters were blinded to the group assignment. The participants in the active arm received tDCS (anodal; F3, cathodal, Fp2, 2A, 20 min) twice daily for five consecutive days, whereas those in the sham arm received the same amount of sham-tDCS. Calculation and reading tasks were conducted in both arms as a form of cognitive intervention for 20 min during tDCS. The primary outcome was the attrition rate during the trial in the active arm, which is expected to be less than 10%. The secondary outcomes were the between-group differences of adjusted means for several cognitive scales from baseline to post-intervention and follow-up.

**Results:**

Twenty patients [nine women (45%)], with a mean (standard deviation) age of 76.1 years participated; nine patients (45%) with minor neurocognitive disorders and 11 (55%) with major neurocognitive disorders were randomized, and 19 of them completed the trial. The attrition rate in the active arm was 0%, with no serious adverse events. Further, in the Intention-to-Treat analysis, patients in the active arm showed no statistically significant improvement compared with those who received the sham in the mean change scores of the mini-mental state examination [0.41; 95% CI (−1.85; 2.67) at day five, 1.08; 95% CI (−1.31; 3.46) at follow-up] and Alzheimer’s disease assessment scale – cognition subscale [1.61; 95% CI (−4.2; 0.98) at day 5, 0.36; 95%CI (−3.19; 2.47) at follow-up].

**Conclusion:**

These findings suggest that tDCS is safe and tolerable but causes no statistically significant cognitive effects in patients with mild or major neurocognitive disorders. Additional large-scale, well-designed clinical trials are warranted to evaluate the cognitive effects of tDCS as an augmentation to cognitive training.

**Clinical Trial Registration:**

www.ClinicalTrials.gov, identifier NCT03050385.

## Introduction

Dementia is a disorder characterized by cognitive decline that interferes in patients’ daily living and social functioning. Alzheimer’s disease (AD), is the most common cause of developing dementia, and its progression is usually insidious and slow. Often, a prodromal and transitional state exists, without loss of independence, called mild cognitive impairment (MCI) (Petersen et al., 1999; Gauthier et al., 2006). The progression of dementia not only causes functional impairment in patients, it also degrades the caregiver’s quality of life or social functioning due to caregivers’ burden (Gill et al., 2017). Currently approved pharmacotherapies, cholinesterase inhibitors, and memantine are not disease-modifying and cannot revert the course of the disease, although they show a slight improvement in cognitive scales (Birks, 2006). Therefore, increasing focus has been placed on delaying deterioration or conversion from MCI to AD and other forms of dementia.

Recent studies have gradually revealed a few potentially modifiable factors, such as physical inactivity, social isolation, and depression (Gill et al., 2017). Further, a few cognitive interventional studies have also been conducted. Cognitive training generally includes guided practice on a set of standardized tasks designed to reflect specific cognitive domains. A recent meta-analysis indicated that the overall effect of cognitive training on cognition in MCI was moderate (Hedges’ *g* = 0.35) and that in dementia was small (*g* = 0.26) (Hill et al., 2017). However, many of these trials assessed short-term cognitive outcomes. Moreover, based on the results from randomized trials that lasted for at least 6 months, cognitive training in patients with MCI suggested no effects on performance, with low strength, and insufficient evidence (Butler et al., 2018). Therefore, further strategies are awaited to combat cognitive decline in such patients. Transcranial direct current stimulation (tDCS) is a non-invasive neuromodulation technique, which involves passing a direct electrical current through the cerebral cortex, usually *via* two electrodes placed on the scalp. One electrode serves as an anode and the other functions as the cathode. The tDCS device generates and delivers a small electric current (usually 1 to 2 mA) to different areas of the brain (Yokoi et al., 2018). The basic mechanism of tDCS is that the anodal tDCS increases neuronal excitability by causing a depolarization of the resting potential, while the cathodal tDCS hyperpolarizes the resting potential, thereby suppressing neuronal excitability (Philip et al., 2017). The change in neuronal excitability may lead to alteration in brain functioning in the vicinity of the stimulated area (Meinzer et al., 2015). Further, the alteration in brain functioning may also be explained by the hypothesis that prolonged membrane polarization by tDCS changes neuroplasticity through N-Methyl-D-aspartic acid (NMDA) receptors, thereby leading to long-lasting aftereffects of tDCS (Nitsche et al., 2003).

A recent meta-analysis of randomized controlled trials indicated that tDCS may be effective on cognition in healthy participants (Dedoncker et al., 2016); however, a meta-analysis of randomized controlled trials on the effect of tDCS on cognition in patients with dementia and MCI shows that tDCS is not always effective overall (Inagawa et al., 2018). The discord among the findings of these studies may be due to differences in the electrode montage, stimulation parameters, and timing of tDCS in the training tasks (Liu et al., 2017). Further, according to our systematic review and meta-analysis (Inagawa et al., 2018), only one study among the 11 previous studies on the effects of tDCS on cognition in dementia and MCI described a plan to provide sample size calculation. In addition, although we had planned to estimate sample size from previous studies, no study assessed the effect of tDCS combined with cognitive rehabilitation in patients with MCI at July 2016, when we started this study. Therefore, it was impossible to calculate sample size from the results of previous studies. It is important to provide *a priori* sample size calculation in order to gain sufficient statistical power to detect a difference in clinical trials. However, many previous studies did not provide sample size calculations and, thus, the results from these studies may be false-negatives, when the results are actually positive. Further, according to our meta-analytic review (Inagawa et al., 2018), the quality of study designs in these previous studies seems to be poor. In fact, many of them did not clearly state allocation concealment, blinding of personnel, or any method of handling missing data. These problems may overestimate the effects of tDCS, although tDCS is actually not effective.

Further, previous studies on tDCS revealed the beneficial cognitive/anti-depressant effects of anodal tDCS in AD patients undergoing simultaneous cognitive training (Hsu et al., 2015), in depressive patients taking antidepressants (Brunoni et al., 2013), and in MCI patients receiving physical therapy (Manenti et al., 2016). Further, a randomized trial demonstrated that active tDCS, but not sham, over DLPFC combined with a working memory task showed greater improvement in healthy participants in terms of the performance of an attention and working memory test 1 month after the final treatment, compared with tDCS alone (Martin et al., 2013). These studies indicate the possibility of augmentation strategies of tDCS simultaneously with the conduct of cognitive training for improving cognition in patients with dementia and MCI.

The objectives of the proposed study are to assess the safety and feasibility of tDCS during cognitive rehabilitation, as well as to estimate potential efficacy applicable for further confirmatory studies in patients with neurocognitive disorders. Because combining tDCS with cognitive training may enhance the benefits of tDCS, we hypothesize that tDCS will improve, particularly when administered during cognitive tasks in patients with these disorders.

## Materials and Methods

### Trial Design

This exploratory study was a single-arm study in terms of safety, while this was a two-arm, parallel, randomized, sham-controlled trial for the assessment of potential efficacy, and feasibility among 20 participants with a diagnosis of major neurocognitive disorder or mild neurocognitive disorder based on the Diagnostic and Statistical Manual of Mental Disorders (DSM-5) (American Psychiatric Association, 2013). This study is reported in accordance with the CONSORT (Consolidated Standards of Reporting Trials) 2010 statement of information to include when reporting a randomized trial, which was developed to provide guidance in the form of a checklist of recommended items to help improve the quality of a study design (Moher et al., 2010; Supplementary Table S1).

Participants were supposed to be randomly assigned in the ratio of 1:1 to one of two groups – an active group or a sham group – using the order of entry into the study and a computer-generated randomization list obtained using a computer-generated randomization method, MUJINWARI (IRUKA System Cooperation, Tokyo, Japan). This was done to ensure a balanced allocation of the following factors across groups: age range (55–60 years, 61–70 years, 71–80 years, or 81–90 years), sex (male or female), and diagnosis (major neurocognitive disorder or mild neurocognitive disorder). This randomization method includes stratification for all of those factors. The allocation sequence was concealed until they completed the follow-up evaluations. tDCS device was always kept at the back of participants’ visibility during its administration so that participants did not recognize allocation concealment. tDCS administrators only obtained access to a computer-generated randomization list, and tDCS administrators kept the allocation secret so that outcome raters did not recognize allocation concealment. Both the participants and raters were blinded to the group allocation; however, the investigators and those administering the tDCS were not blinded to the group allocation. In order to assess the quality of the blinding, after completing the study on day 5, the participants were asked to guess whether they were allocated to the active or sham group of the study. Further, we evaluated demographic and clinical characteristics, and used these data to provide descriptive characteristics of the population, and to analyze whether these characteristics could predict the outcomes. This study was registered on ClinicalTrials.gov (NCT03050385).

### Participants

Both inpatients and outpatients were recruited by referrals from psychiatrists in a single academic hospital: National Center of Neurology and Psychiatry in Tokyo, Japan; both male and female patients were selected. The principal investigators or sub investigators assigned participants to interventions. The following were the key inclusion criteria: (a) subjects aged between 55 and 90 years and diagnosed with either major neurocognitive disorder or mild neurocognitive disorder, as defined in DSM-5; (b) subjects taking a stable dose of anti-dementia medications, such as cholinesterase inhibitors or memantine, for at least 2 weeks preceding enrollment; and (c) subjects who are able to walk independently, with or without an aiding device. The following were the key exclusion criteria: (a) subjects with severe psychotic symptoms requiring antipsychotic treatment, (b) subjects estimated to be in need of hospitalization within 6 weeks because of severe depression and/or suicidal ideation, (c) subjects who have a clinical contraindication to electroconvulsive therapy or tDCS, (d) subjects with an MMSE score less than 18 or a clinical dementia rating-Japanese version (CDR-J) global score of more than two, (e) subjects who were unable to attend more than 2 days of the trial, and (f) subjects for whom MMSE subscales of either “write a sentence” or “copy a figure” was zero. The patients were carefully assessed by a specialized psychiatrist before the trial. Because of the safety of tDCS to date (Bikson et al., 2016), no specific exclusion criteria were applied. Patients who were receiving anti-dementia medications were not excluded, but they were required to be receiving stable doses of these medications for at least 2 weeks prior to the first day of the administration of stimulation.

### Intervention

Transcranial direct current stimulation was performed using a specially developed battery-driven constant 1 × 1 low-intensity tDCS (Model 1300A; Soterix Medical Inc., New York, NY, United States) that delivers direct current through two saline-soaked surface sponge electrodes (35 cm2) with a maximum output of 2 mA. This device also has a switch off allocation. If the administrator turned the switch on, the sham stimulation was delivered; if the administrator turned the switch off, active stimulation was delivered. During the stimulation, the device was placed behind the participants, and their allocation was kept secret so that they would not know which group they were randomized to. The anode electrode was placed over the left dorsolateral prefrontal cortex (DLPFC) (F3) and the cathode was placed over the contralateral supraorbital ridge (Fp2), using the 10/20 electrode placement system in both active and sham arms. This method of DLPFC placement has been established by neuro-navigation techniques, as this method is relatively accurate for localization (Herwig et al., 2003). The DLPFC was selected because a previous study on healthy participants indicated that tDCS over DLPFC had beneficial effects on working memory (Martin et al., 2013). The cathode electrode was placed over the contralateral supraorbital area, which was similar to recent tDCS studies on cognitive functioning in patients with MCI (Manenti et al., 2016), Alzheimer’s dementia (Khedr et al., 2014), and schizophrenia (Narita et al., 2017). It must be noted that for aging populations, stimulation with 2 mA has been shown to be safe. Actually, no severe adverse events due to tDCS with 2 mA have been reported (Bikson et al., 2016). The participants in the active arm received active tDCS at a constant current with an intensity of 2 mA for 20 min per session, with two sessions per day for 5 consecutive days. Those in the sham arm received the same treatment as those in the active arm, but the overall active stimulation period was only 60 s, including the 30 s for both the fade-in and fade-out periods. For the other periods, the stimulator remained active but did not generate current for 20 min in each session. Therefore, those in the sham arm usually experienced an initial itching sensation but received no current for the remainder of the session. All the participants received both tDCS (either active or sham) and cognitive training task for 20 min per session. On each day, the second active or sham tDCS session was conducted at least 20 min after the end of the first tDCS session in order to take into consideration the aftereffects of tDCS. In other words, the interval between the first and second tDCS session was 20 min.

**FIGURE 1:**
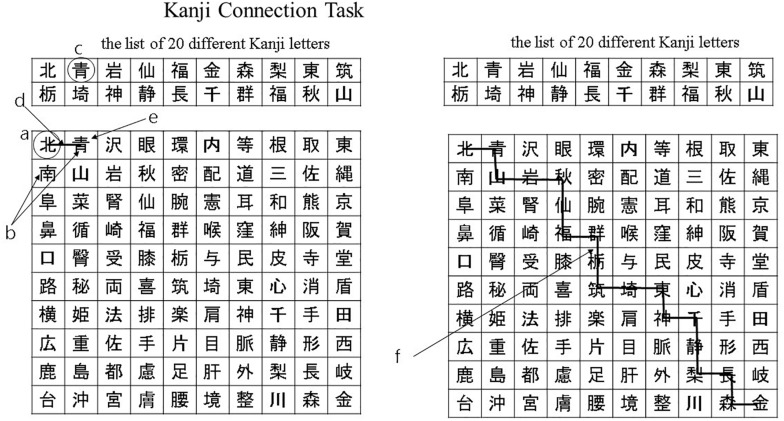
Kanji connection task. **(a)** The participants began from the first Kanji letter in the upper-left corner of the 10 × 10 grid. **(b)** The participants were instructed to look at the Kanji letters on the right and toward the bottom of the first Kanji letter. **(c)** The participants were instructed to match it with a Kanji letter included in the list of 20 different Kanji letters in the table. **(d)** When one of them would match, they connected this Kanji letter to the first Kanji by drawing a line from one to another. **(e)** The new Kanji letter would take the place of the first Kanji letter. **(f)** The participants were asked to repeat this process for all the Kanji letters until they reached the Kanji letter in the bottom-right corner of the grid.

These tasks comprised an initial 10 min calculation task, followed by a 10 min language task in Japanese. During the calculation task, participants solved basic arithmetic questions – such as single-digit addition, subtraction, and multiplication – as quickly and accurately as possible. During the language tasks, which included the Kanji writing task and the Kanji connecting task, participants answered questions related to Japanese Kanji letters. All the questions were printed on A4 sheets (210 mm × 297 mm). In the Kanji writing task, each participant was asked to interpret the meaning of hiragana characters and to write letters in Kanji. The participants performed a Kanji connecting task on a 10 × 10 grid, which contained a Kanji letter in each grid. In a separate table, there was a list of 20 different Kanji letters. In this task, the participants began from the first Kanji letter in the upper-left corner of the 10 × 10 grid (Figure 1a). Next, the participants were instructed to look at the Kanji letters on the right and toward the bottom of the first Kanji letter (Figure 1b) and match it with a Kanji letter included in the list of 20 different Kanji letters in the table (Figure 1c). When one of them would match, they connected this Kanji letter to the first Kanji by drawing a line from one to another (Figure 1d). In the next step, the new Kanji letter would take the place of the first Kanji letter (Figure 1e). The participants were asked to repeat this process for all the Kanji letters until they reached the Kanji letter in the bottom-right corner of the grid (Figure 1f).

The difficulty and complexity of the cognitive tasks were the same across sessions, but the content was different. We did not calculate the scores that could be obtained from the tasks. A previous randomized controlled trial indicated that similar working memory tasks (reading and simple arithmetic problems) as the one in our pilot study improve executive functions, verbal episodic memory, focus attention, and processing speed in elderly patients who are healthy (Nouchi et al., 2016). The rationale for using these working memory tasks is that the training activates the bilateral prefrontal cortex (Arsalidou and Taylor, 2011). We also chose this task because the task was familiar to Japanese elderly population and feasible to be conducted among patients with dementia (Kawashima et al., 2005). We were interested in investigating the effects of tDCS on global cognition when combined with the cognitive training task commonly used for the elderly population in Japan, in order to ensure whether clinically meaningful effects can be obtained by adding multisession tDCS to cognitive training using common cognitive measures of MMSE and ADAS-Cog.

### Outcomes

As this was the first phase of an exploratory feasibility trial, a sample size of 20 individuals, including sham, was adapted. In the intervention group, the primary outcome was the attrition rate during the trial, which is expected to be less than 10%. We selected the attrition rate as the primary outcome because it was important to first assess the safety and feasibility of tDCS in Japanese patients with neurocognitive disorders due to the fact that, to the best of our knowledge, no previous studies were found in such patients receiving tDCS while simultaneously being engaged in cognitive rehabilitation in Japan on July, 2016 when we started this study. The secondary outcomes were between-group differences in mean ADAS-Cog, MMSE, frontal assessment battery (FAB), and CDR-J scores from the baseline. We selected ADAS-Cog (whose score ranged from 0 to 70) and MMSE (whose score ranged from 0 to 30) for the assessment of global cognition, FAB (whose score ranged from 0 to 18) for the evaluation of frontal lobe functions, and CDR (whose score ranged from 0 to 3) for the estimation of the severity of dementia. All the above-mentioned outcome measures were scored by a psychologist after a clinical interview, who was blinded to group allocation. The outcome measures were assessed at the baseline, at the end of the final stimulation, and 2 weeks after the final stimulation (Supplementary Table S2).

### Data Collection Methods and Data Monitoring

The assessments were conducted at the baseline, immediately after the intervention, and 2 weeks after the end of the intervention (Supplementary Table S2). Baseline and follow-up evaluations were conducted by experienced psychologists, who were blinded to the group assignments. The outcome data was sent to an independent data monitor, and neither the investigators nor the raters handled any data directly throughout the study. The data were initially recorded on paper files, with each participant assigned to a code number. These files were stored in a locked security box. Upon completion of the follow-up data collection, the data was sent to an independent data tester for cleaning up. The data monitor center also oversaw and reviewed the progress of the trial. If a participant decided to withdraw their consent, we allowed that participant to stop at any time. We also ceased the intervention if we observed any severe adverse events like burning. In this pilot study, the Efficacy and Safety Assessment Committee, which comprised members that were independent of the research, in the National Center of Neurology and Psychiatry checked and assessed whether or not this clinical trial was conducted safely and appropriately. The committee was called upon to decide whether it is possible to continue the trial or whether the research protocol must be revised in cases of either severe adverse events or protocol violations. The committee also performed this procedure by checking the documents of this trial in the intermediate period when five participants completed or discontinued their participation in this trial. The safety questionnaire on adverse events was established at the time according to the guidelines published in a recent consensus paper (Brunoni et al., 2011).

### Statistical Analysis

We conducted an intention-to-treat analysis for patients who were randomized to either the active or sham arms; in addition, we summarized demographic data for all patients. Further, we calculated the point estimate of tDCS-related dropout proportion in the intervention group, where we checked whether the estimate was less than 10%. The exact confidential intervals (CIs) of this binomial proportion (Clopper and Pearson, 1934) were assessed. In order to evaluate the mean treatment effect, we conducted mixed models for repeated measures (MMRM) analysis to detect changes from baseline in ADAS-Cog, MMSE, FAB, and CDR-J at day 5 and follow-up. The MMRM analysis models included the covariates of age (55–60 years, 61–70 years, 71–80 years, or 81–90 years), sex (male or female), and disease (dementia or MCI), which were the stratification factors of dynamic allocation. This MMRM analysis models had treatment groups, time, group-by-time interaction, age, sex, diagnosis, and baseline as a fixed effect and unstructured covariance structure. Fisher’s exact test was used to assess the integrity of blinding. We used STATA 14 (StataCorp LP, College Station, TX, United States) and SAS version 9.4 to conduct the statistical analysis.

## Results

### Participants

**FIGURE 2:**
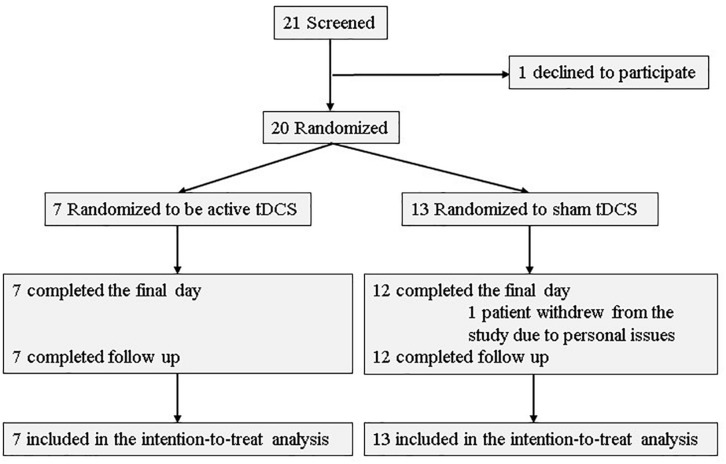
Flow chart of participant selection.

Of the 21 participants who agreed to provide written consent, 20 were randomized to either the active or sham arms. Seven patients (five patients had AD, one had lewy body disease (LBD), and the other had the other type of etiology) were allocated to the active arm, while 13 patients (11 patients had AD, one had LBD, and the others had unspecified types of neurocognitive disorders) were allocated to the sham arm. Further, 19 participants (seven in the active arm and 12 in the sham arm) received all 10 tDCS sessions and completed the final assessment. All patients in tDCS group were outpatients. Two participants withdrew from the study: one withdrew during the intervention phase, and one before randomization. Figure 2 depicts a flow chart on participants’ selection, and Table 1 presents baseline characteristics. Recruitment and follow-up were conducted from July 2016 until July 2017 because more than 20 participants finished follow-up examinations. 20 participants were included for statistical analysis.

**TABLE 1:** Demographics and clinical characteristics (*n* = 20).

	**Active group**	**Sham group**
	**Mean ± SD or n (%)**	

	7 (100%)	13 (100%)
Age (year)	76.6 ± 5.7	76.2 ± 7.7
Female	4 (57.1%)	6 (46.2%)
Major neurocognitive disorder	3 (42.9%)	7 (53.8%)
Right-handed	7 (100%)	13 (100%)
Duration since diagnosis (year)	0.9 ± 1.2	1.2 ± 1.5
Family history		
Dementia	3 (42.9%)	3 (23.1%)
Mental disorder	0 (0%)	0 (0%)
Neurological disorder	1 (14.3%)	1 (7.7%)
Medication over the past 6 months		
Antidepressant, antipsychotics	1 (16.7%)	3 (23.1%)
Benzodiazepine	3 (42.9%)	4 (30.8%)
Cholinesterase inhibitors	4 (57.1%)	10 (76.9%)
Past history		
Substance abuse disorder	1 (14.3%)	0 (0%)
Schizophrenia	0 (0%)	0 (0%)
Mood disorder	0 (0%)	0 (0%)
Neurologic disorder	0 (0%)	2 (15.4%)
Head trauma	1 (14.3%)	2 (15.4%)
Visits to day care center for seniors	1.3 ± 2.6	0 ± 0

*SD, standard deviation.*

### Primary Outcome

The primary outcome was an attrition rate in the intervention group. The attrition rate in the group was 0%, with no requirements of hospitalization, trial discontinuation, or any specific treatment in the active arm. The CIs of this proportion were from 0.0 to 41.0%.

### Secondary Outcomes

#### MMSE

**FIGURE 3:**
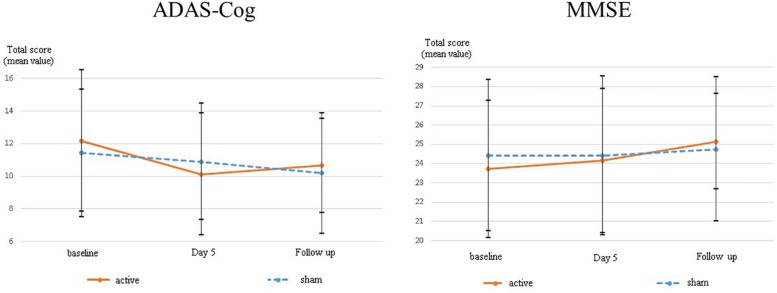
The change scores in adjusted mean difference from baseline on ADAS-Cog and MMSE. In order to understand the mean change from baseline in each group easily, baseline scores in each group were shown as zero in this graph.

The differences in adjusted means between groups for MMSE scores were 0.41 [95% CI: −1.85 to 2.67] (*p* = 0.705) at day five, and 1.08 [95% CI: −1.31 to 3.46] (*p* = 0.352) at follow-up, respectively (Table 2). There was no statistical significance in the between-group difference between the active and sham groups. Supplementary Table S3 presents the change scores in adjusted mean difference from the baseline in each group. Figure 3 illustrates the mean values and standard deviations (SDs) at each point.

**TABLE 2:** The differences in adjusted means between groups for each cognitive scale in the MMRM analysis.

	**Active tDCS vs. Sham**							
**Clinical**	**Post-treatment**				**Two-weeks follow**			
**studies**	**from baseline**				**up from baseline**			
		**95%**				**95%**		
	**Difference**	**CI**		***p***	**Difference**	**CI**		***p***
MMSE	0.41	−1.85	2.67	0.705	1.08	1.31	3.46	0.352
ADAS-Cog	−1.61	−3.19	2.47	0.205	−0.36	−3.19	2.47	0.791
FAB	−2.27	−6.17	1.63	0.233	−3.01	−6.46	0.45	0.083
CDR					0.06	−0.09	0.22	0.404

*MMRM, mixed-effect model repeated measurement; tDCS, transcranial direct current stimulation; 95% Cl, 95% confidential intervals; MMSE, mini-mental state examination; ADAS-Cog, Alzheimer dementia assessment scale – cognitive subscale; FAB, frontal assessment battery; CDR-J, clinical dementia rating-Japanese version.*

#### ADAS-Cog

The differences in adjusted means between groups for ADAS-Cog scores were 1.61 [95% CI: −4.2 to 0.98] (*p* = 0.205) at day five and 0.36 [95% CI: −3.19 to 2.47] (*p* = 0.791) at follow-up (Figure 3). There was no statistically significant difference between the active and sham arms. Supplementary Table S3 presents the change scores in the adjusted mean difference from baseline in each group. Figure 3 illustrates the mean values and SDs at each point.

#### FAB

The differences in adjusted means between groups for FAB scores were −2.27 [95% CI: −6.17 to 1.63] (*p* = 0.233) at day five and −3.01 [95% CI: −6.46 to 0.45] (*p* = 0.083) at follow-up. FAB showed a dip in the active arm at both day five and follow-up, but no statistically significant differences were found between the groups. Supplementary Table S3 presents the change scores in the adjusted mean difference from baseline in each group.

#### CDR

The differences in adjusted means between groups for CDR scores were 0.06 [95% CI: −0.09 to 0.22] (*p* = 0.404) at follow-up. Supplementary Table S3 presents the change scores in the adjusted mean difference from baseline in each group.

### Adverse Events

We found neither severe adverse events nor the need for medications caused by adverse events in each group. Table 3 presents adverse events related to tDCS.

**TABLE 3:** Adverse effects related to tDCS reported by patients in each group.

	**tDCS**	**Sham**	***p***
Headache (n, %)	1 (14.3%)	5 (38.5%)	0.354
Neck pain (n, %)	0 (0%)	2 (15.4%)	0.521
Scalp pain (n, %)	0 (0%)	4 (30.8%)	0.249
Tingling (n, %)	3 (42.9%)	9 (69.2%)	0.428
Itching (n, %)	1 (14.3%)	3 (30.8%)	1.000
Burning sensation (n, %)	2 (28.6%)	4 (30.8%)	1.000
Skin redness (n, %)	2 (28.6%)	2 (15.4%)	0.587
Sleepiness (n, %)	1 (14.3%)	2 (15.4%)	1.000
Trouble concentrating (n, %)	1 (14.3%)	1 (7.7%)	1.000
Acute mood change (n, %)	0 (0%)	1 (7.7%)	1.000
Others (n, %)	0 (0%)	0 (0%)	1.000

### Integrity of Blinding

In the sham and active groups, seven of 12 participants (58.3%) and three of seven participants (42.9%), respectively, correctly identified the allocation group (*p* = 1.000, as assessed by Fisher’s exact test). Thus, participants were unable to guess their actual group beyond that by chance.

## Discussion

Using data from a small sized sample, no cognitive effects of tDCS were detected in this pilot study; however, this is certainly not definitive because of the insufficient sample size used in this study. Further studies using a larger sample size are warranted in order to arrive at a clear conclusion regarding whether or not tDCS is effective for improving cognition in patients with mild or major neurocognitive disorders and also to evaluate the generalizability of this pilot study. Moreover, we did not adjust for multiplicity because the primary objective of this study was to assess the safety and feasibility of tDCS in Japanese elderly patients with neurocognitive disorders, and the secondary aim was to exploratorily estimate potential efficacy applicable for further proof-of-concept studies to demonstrate the effects of tDCS on cognition in patients with neurocognitive disorders. Apart from that, the augmentation strategy in this pilot study should have been more sophisticated. Although proof of concept has been indicated, whereby anodal stimulation over DLPFC during a working memory task led to enhanced performance the next day (Martin et al., 2013), the cognitive training task used in this pilot study is not exactly the same as that employed in previous studies. Moreover, the Kanji connection task is short and tough as compared with that in previous studies. A previous study indicates that those trainees who put more effort into training were more anxious or depressed, and showed lesser improvement in cognition (McAvinue et al., 2013). Therefore, it may be necessary not only to modify the tDCS protocol but also to optimize the cognitive task in future trials.

Further, the target population may not have been optimal. A previous randomized controlled trial indicates that tDCS is not effective on global cognition assessed by ADAS-Cog in patients with moderate and severe dementia with apathy. The authors discuss that in order to gain cognitive benefits of anodal repetitive tDCS, it is necessary to have at least some remaining neuronal function to promote plasticity, which may not be possible in aged patients with AD. In this study, the target population may have been patients in an early stage of the disease, like MCI. Moreover, the difference between mild neurocognitive disorders and major neurocognitive disorders is the interference with independence in daily activities. Then, cognitive deficits in mild neurocognitive disorders may be stable or even reversible, but those in major neurocognitive disorders may be continuous or even progressive. These differences between the mild and major disorders especially for the potential difference in the course of cognitive change may have confounded the results presenting the after-treatment cognitive changes from baseline although we did include the variable as a covariate in the analysis. A preliminary randomized sham-controlled trial indicated that comparisons of anodal tDCS for the DLFPC group (2 mA, 25 min daily for 10 days) vs. the sham group revealed significant interaction between time and treatment for MMSE scores post-stimulation, 1 month later, and 2 months later in 22 patients (11 in each group) with mild-to-moderate Alzheimer’s dementia (*p* = 0.04) (Khedr et al., 2014). Another preliminary randomized sham-controlled trial indicated that anodal tDCS over the DLPFC group (2 mA, 25 min daily for 10 days) vs. the sham group showed a significant interaction between time and treatment (*p* = 0.0041) on the Parkinson’s disease cognitive rating scale at the post-stimulation point and 3-month follow-up period in 20 patients (10 in each group) with MCI in Parkinson’s disease (Manenti et al., 2016). On the other hand, no significant effect of anodal tDCS over DLPFC was found on the Apathy scale (*p* = 0.55 for repeated measures) or ADAS-Cog (*p* > 0.40) in 40 patients (20 in each group) with moderate-to-severe AD compared to the sham group (Suemoto et al., 2014). Moreover, tDCS may not be effective in global cognition in these patients. Although a few studies show potential cognitive benefits, a functional trade-off has been suggested in which improvement in a single cognitive domain comes at the cost of decline in another one (Philip et al., 2017). In addition, the effect of tDCS appears to be site-specific; thus, the effect of tDCS in itself may not sufficiently transfer to other brain regions to improve global cognition (Kim et al., 2014).

In order to determine whether or not the effect of tDCS on cognition in neurocognitive disorders is clinically meaningful, one of the possible options is to compare the effect of tDCS with that of the first-line standard treatment. Although Cholinesterase inhibitors (ChE-Is) have never been approved for standard treatment in patients with MCI in Japan, they are considered to be the first-line pharmacological agent for mild to moderate AD. ChE-Is work by inhibiting the breakdown of acetylcholine, an important neurotransmitter related to memory, by blocking the enzyme acetylcholinesterase. The between-group difference in mean changes of MMSE was 1.37, according to a systematic review and meta-analysis of unconfounded, double-blinded, randomized, placebo-controlled trials designed to evaluate the efficacy of patients with dementia due to AD, in which treatment with a ChE-I was administered for approximately 6 months (Birks, 2006). If the effect size obtained from the ongoing phase-II randomized trial is similar to that in our pilot study, which was 1.08 at follow-up, tDCS could be a potential tool for alleviating cognitive deficits in those patients. Further, the differences in adjusted means between groups for ADAS-Cog scores at day five was −1.61 (*p* = 0.205) (Figure 3). Further trials are warranted to evaluate whether these cognitive benefits can be generalized to the larger population in MCI and mild dementia in this tDCS protocol.

The strength of this study is that it has a relatively low bias risk as compared with previous studies. Although tDCS administrators were not blinded, both participants and raters were blinded. A random sequence was generated through computers, and allocation was concealed until the disclosure of the data; blinding was well integrated. Further, all pre-specified outcomes were shown after registration on ClinicalTrials.gov. The dropout rates were low in both groups: 0% in the active group and 7.69% in the sham group. These indicate the quality of this study. Another strength of our study is the novelty that, to the best of our knowledge, this is the first study to assess optimized tDCS protocol combined with cognitive training in patients with both MCI and mild dementia. Further, our pilot study indicated that tDCS combined with cognitive training was safe and feasible. In addition, we selected ADAS-Cog and MMSE because these scales are the screening tools that are most commonly used to measure cognitive deficits in clinical settings.

This study has a few additional limitations. First, the follow-up period is too short to evaluate changes in disease progression and to test whether additional interventions are needed over time. Second, cognitive training tasks used in this pilot study are not entirely the same as those used in previous studies, which indicates that calculation tasks and reading tasks improve executive functions, verbal episodic memory, focus attention, and processing. Third, our cognitive training protocol is short and tough compared to that of previous studies. This may have caused psychological stress among participants, which may have decreased the effect of cognitive training (McAvinue et al., 2013). Fourth, our pilot study only selected MMSE and ADAS-Cog total scores for the assessment of global cognition and FAB for the evaluation of frontal lobe cognitive function. In future studies, a standard scale, like the repeatable battery for the assessment of neuropsychological status (RBANS), is an appropriate choice to comprehensively assess the global cognition and cognitive domains separately and to gain statistical power enough to assess the meaningful cognitive benefits in patients with early stages of neurocognitive disorders. For example, RBANS may be well suited because it is a brief and comprehensive battery consisting of the following indices: memory, attention, language, visuospatial/constructional, and total scores. This scale enables raters to simultaneously assess global cognition and several other domains of cognition, including memory, attention, and language. Fifth, we selected MMRM analysis for multiple outcome measures and several time points, so the results obtained from those measures should be interpreted carefully. Future trials with a proper sample size calculation for one primary outcome may provide meaningful results. Sixth, a small sample size may lead to lack of statistically significant power. It is important to provide *a priori* sample size calculation in order to gain sufficient statistical power to detect a difference in clinical trials. However, we did not provide it because it was necessary to first assess the safety and feasibility of tDCS in Japanese patients with neurocognitive disorders, as, to the best of our knowledge, this is the first pilot study of tDCS in such patients. Therefore, the results obtained from this study may well lack statistical power to detect a difference between the active and sham arms. If the primary outcome of a future trial lies in the differences of adjusted means between groups for ADAS-Cog scores at day five, the minimal sample size with statistical power over 0.8 is estimated to be 46 in each group; this is based on the assumption that the between-group difference in ADAS-Cog scores is −1.61 and its SD is 2.70. Based on this sample size calculation, we have initiated a phase-II randomized, sham-controlled trial of tDCS on cognition in MCI and mild dementia. This trial has been registered in the Japan Registry of Clinical Trials (protocol number: jRCTs032180016). Seventh, the patients were supposed to be randomized to the treatment groups with a 1:1 ratio but the outcome of the allocation was 7:13. That was because too many factors were included in the factors for minimization techniques in spite that the sample size was small. Eighth, the results may be interpreted carefully because we conducted statistical tests for a variety of outcome groups without taking multiplicity into consideration because this trial was an exploratory trial.

In conclusion, tDCS is safe and tolerable in the context of cognitive rehabilitation. We found no statistically significant cognitive effects of tDCS in patients with mild or major neurocognitive disorders. Further trials with larger samples may clarify the efficacy of tDCS on global cognition and several cognitive domains in such patients.

## Ethics Statement

This study was conducted in accordance with the Declaration of Helsinki and is based on the Ethical Guidelines for Medical and Health Research Involving Human Subjects in Japan. The protocol was presented to the institutional review board of the National Center of Neurology and Psychiatry Ethics Committee for approval (A2016-048). The principal investigator and the research coordinator were responsible for conducting the informed consent process with all the study participants. All the participants were required to provide written consent to participate in the trial. They were assessed only after being informed of the objectives of the study and providing written informed consent. Any relevant changes in the study protocol and/or the informed consent process were sent to the Ethical Committee as protocol amendments. The identities of all the participants were protected using individual codes, which were accessible only by the principal investigator. The protocol was also registered on ClinicalTrials.gov.

## Author Contributions

TI and YY developed the original concept of the trial. ZN and KN advised and reviewed the design and methodology of the original protocol. YY established the protocol. TI and ZN conducted the tDCS. KM conducted the statistical analysis of the results. TI wrote the manuscript and all other authors reviewed and commented on the draft. MO contributed to the safety monitoring and management of our research progress. All authors read and approved the final manuscript.

## Conflict of Interest Statement

The authors declare that the research was conducted in the absence of any commercial or financial relationships that could be construed as a potential conflict of interest.

## Supplementary Material

TABLE S1 CONSORT statement.

TABLE S2 Study Procedure. The outcome measures were assessed at the baseline, at the end of the final stimulation, and two weeks after the final stimulation.

TABLE S3 The change scores in adjusted mean difference from baseline in each group.

## Abbreviations

AD: Alzheimer’s disease
ADAS-Cog: Alzheimer dementia assessment scale – cognitive subscale
CDR-J: clinical dementia rating-Japanese version
CI: confidential interval
DLPFC: dorsolateral prefrontal cortex
FAB: frontal assessment battery
LBD: lewy body disease
LTP: long-term potentiation
MCI: mild cognitive impairment
MMRM: mixed-effect model repeated measurement
MMSE: mini-mental state examination
SD: standard deviation
tDCS: transcranial direct current stimulation.
