# Supplementary material for: Safety and Feasibility of Transcranial Direct Current Stimulation for Cognitive Rehabilitation in Patients With Mild or Major Neurocognitive Disorders: A Randomized Sham-Controlled Pilot Study
Source: Front Hum Neurosci. 2019 Sep 6;13:273. doi: 10.3389/fnhum.2019.00273 (PMC6742726; doi:10.3389/fnhum.2019.00273)
Supplement: TABLE S2 — Study Procedure. The outcome measures were assessed at the baseline, at the end of the final stimulation, and two weeks after the final stimulation. [file Table_2.DOCX]

**Supplementary Table 2**

|  | period | | | |
| --- | --- | --- | --- | --- |
|  | Screening | Day 1 | Day 5 | Follow up (3 weeks after day 5) |
| Timing of session | Within 14 days prior to Day 1 |  |  | ±7 days |
| Demographics | ✔ | ✔ |  |  |
| Clinical Dementia Scale | ✔ |  |  |  |
| Physical examination | ✔ | ✔ |  |  |
| Recording of adverse events |  | （all stimulation days）  ✔ | | ✔ |
| ADAS-cog | ✔ |  | ✔ | ✔ |
| Calculation task |  | ✔ | ✔ | ✔ |
| MMSE | ✔ |  | ✔ | ✔ |
| FAB | ✔ |  | ✔ | ✔ |
| CDR-J | ✔ |  |  | ✔ |
| Assessment of blinding |  |  | ✔ |  |
