# Supplementary material for: Safety and Feasibility of Transcranial Direct Current Stimulation for Cognitive Rehabilitation in Patients With Mild or Major Neurocognitive Disorders: A Randomized Sham-Controlled Pilot Study
Source: Front Hum Neurosci. 2019 Sep 6;13:273. doi: 10.3389/fnhum.2019.00273 (PMC6742726; doi:10.3389/fnhum.2019.00273)
Supplement: TABLE S3 — The change scores in adjusted mean difference from baseline in each group. [file Table_3.DOCX]

Supplementary Table 3. The change scores in adjusted mean difference from baseline in each group.

|  | active tDCS plus cognitive training | | |  |  |  | sham tDCS plus cognitive training | | |  |  |  |
| --- | --- | --- | --- | --- | --- | --- | --- | --- | --- | --- | --- | --- |
| Clinical scales | post-treatment from baseline |  |  | 2-week follow up from baseline |  |  | post-treatment from baseline |  |  | 2-week follow up from baseline |  |  |
|  | mean | SE | p | mean | SE | p | mean | SE | p | mean | SE | p |
| ADAS-Cog total | -2.07 | 0.97 | **0.048** | -1.54 | 0.165 | 0.547 | -0.46 | 0.74 | 0.165 | -1.18 | 0.81 | 0.164 |
| MMSE total | 0.38 | 0.84 | 0.66 | 1.38 | 0.89 | 0.141 | -0.03 | 0.64 | 0.961 | 0.3 | 0.68 | 0.663 |
| FAB total | -0.37 | 1.44 | 0.802 | -0.94 | 1.97 | 0.473 | 1.9 | 1.1 | 0.106 | 2.07 | 0.97 | 0.052 |
| CDR |  |  |  | -0.01 | 0.06 |  |  |  |  | -0.08 | 0.04 |  |

Abbreviation; ADAS-Cog, Alzheimer’s disease assessment Scale- Cognition; MMSE, Mini-Mental State Examination; FAB, Frontal Assessment Battery; CDR, Clinical Dementia Rating; SE, Standard Error.
